# Long-term safety and efficacy of subcutaneous pasireotide in patients with Cushing’s disease: interim results from a long-term real-world evidence study

**DOI:** 10.1007/s11102-019-00984-6

**Published:** 2019-08-22

**Authors:** Luca Manetti, Timo Deutschbein, Jochen Schopohl, Kevin C. J. Yuen, Michael Roughton, Ulrike Kriemler-Krahn, Libuse Tauchmanova, Ricardo Maamari, Carla Giordano

**Affiliations:** 1 Azienda Ospedaliero-Universitaria Pisana, Dipartimento di Medicina Clinica e Sperimentale, Università di Pisa, UO Endocrinologia 2 Ospedale Cisanello Via Paradisa 2, 56124 Pisa, Italy; 2 Division of Endocrinology and Diabetes, Department of Internal Medicine I, University Hospital, University of Würzburg, Würzburg, Germany; 3 Medizinische Klinik und Poliklinik IV, Ludwig-Maximilians-Universität München, Munich, Germany; 4 Swedish Pituitary Center, Swedish Neuroscience Institute, Seattle, WA USA; 5 Barrow Neurological Institute, Phoenix, AZ USA; 6 Novartis Pharma AG, Basel, Switzerland; 7 Novartis Pharmaceuticals Corporation, East Hanover, NJ USA; 8 Section of Endocrinology, Diabetology and Metabolism, Department of Health Promotion Sciences Maternal and Infantile Care, Internal Medicine and Medical Specialities (PROMISE), University of Palermo, Palermo, Italy

**Keywords:** Cushing’s disease, Pasireotide, Hypercortisolism, Pituitary, Safety

## Abstract

**Purpose:**

Clinical trials have demonstrated the favorable efficacy/safety profile of pasireotide in patients with Cushing’s disease (CD). We report interim long-term results of an ongoing real-world evidence study of subcutaneous pasireotide in patients with CD.

**Methods:**

Adults with CD receiving pasireotide, initiated before (prior-use) or at study entry (new-use), were monitored for ≤ 3 years during a multicenter observational study (http://clinicaltrials.gov identifier NCT02310269). Primary objective was to assess long-term safety of pasireotide alone or with other CD therapies.

**Results:**

At the time of this interim analysis, 127 patients had received pasireotide (new-use, n = 31; prior-use, n = 96). Eight patients had completed the 3-year observation period, 53 were ongoing, and 66 had discontinued. Among 31 new-use and 92 prior-use patients with ≥ 1 safety assessment, respectively: 24 (77%) and 37 (40%) had drug-related adverse events (AEs); 7 (23%) and 10 (11%) had serious drug-related AEs. Most common drug-related AEs were nausea (14%), hyperglycemia (11%) and diarrhea (11%); these were more frequently reported in new users and mostly of mild-to-moderate severity. 14 (45%) new-use and 15 (16%) prior-use patients experienced hyperglycemia-related AEs. Mean urinary free cortisol (mUFC) was within normal range at baseline and months 1, 12 and 24, respectively, in: 1/16 (6%), 9/18 (50%), 1/3 (33%) and 0/0 new users; 28/43 (65%), 15/27 (56%), 27/33 (82%) and 12/19 (63%) prior users.

**Conclusions:**

Pasireotide is well tolerated and provides sustained reductions in mUFC during real-world treatment of CD. The lower rate of hyperglycemia-related AEs in prior users suggests that hyperglycemia tends not to deteriorate if effectively managed soon after onset.

**Clinical Trial Registration Number**: NCT02310269.

## Introduction

Cushing’s disease (CD) is a rare, disabling disorder caused by an adrenocorticotropic hormone (ACTH)-secreting pituitary tumor, which triggers overproduction of cortisol by the adrenal glands [1]. Chronic hypercortisolemia is associated with significant morbidity, including metabolic syndrome (comprising hypertension, obesity, diabetes mellitus and dyslipidemia), increased cardiovascular and thromboembolic risk, neurological disorders, infections, and musculoskeletal problems [2]. These complications can substantially impair quality of life and, if untreated, may reduce life expectancy [2].

Pharmacotherapy is an important component in the treatment algorithm for CD and is indicated when pituitary surgery (the first-line approach for most patients) has failed, is not feasible or has been refused by the patient [3–5]. CD persists in 20–30% of patients after surgery, with an additional 25% experiencing disease recurrence after initial remission [1]. As a result, many patients require long-term medication to achieve eucortisolemia and induce disease remission [5]. Long-term, real-world data are therefore required to establish whether the initial efficacy of a given medical therapy is sustained, as well as to identify any safety signals that may evolve over prolonged treatment.

A twice-daily, subcutaneous formulation of pasireotide—a novel second-generation somatostatin analogue [6]—was the first medical therapy to be approved in the USA, Europe and several other countries worldwide to treat patients with CD with persistent or recurrent hypercortisolism after surgery or for whom surgery is not an option [5]. Recently, an intramuscular formulation suitable for once-monthly administration was also approved in the USA, Europe, Japan and Canada [7, 8]. In a large Phase III study in patients with CD, twice-daily pasireotide provided rapid and sustained reduction or normalization of urinary free cortisol (UFC) levels for up to 5 years of treatment [9–11]. Pasireotide has also been shown to improve clinical signs and symptoms of hypercortisolism, reduce cardiometabolic risk factors, and increase health-related quality of life in patients with CD [9–12]. The safety profile of pasireotide was shown to be comparable to that of other somatostatin analogues, except for a higher frequency and degree of hyperglycemia [9–11].

While the efficacy and safety of twice-daily pasireotide have been established in randomized, prospective studies [9–11], few studies have evaluated pasireotide in a real-world clinical practice setting. Herein, we describe interim safety and efficacy results of an ongoing, international, real-world observational study of twice-daily pasireotide in patients with CD (http://clinicaltrials.gov identifier NCT02310269).

## Methods

### Patients

Adult patients (≥ 18 years old) diagnosed with overt CD for whom surgery has failed or is not an option are currently being enrolled in this ongoing study. The diagnosis of CD was made at the discretion of the local investigator. Patients are permitted to receive pasireotide prior to study entry (prior-use group), whereas patients not already receiving pasireotide were initiated at first study visit (new-use group). Planned enrollment for the study is 100–200 patients; the sample size is not based on statistical considerations. Concomitant therapies for CD (e.g. dopamine agonists, steroidogenesis inhibitors) are permitted during the study. Patients are not allowed to participate in the study if they have a cause of Cushing’s syndrome other than CD.

### Study design

This is an ongoing multinational, observational study of pasireotide in patients with CD. Patient enrollment is planned to close in March 2020, with the last patient’s follow-up visit planned for September 2023. To encourage uniform data collection across sites, a suggested schedule of assessments is provided to the investigators; however, specific visit schedules and therapeutic protocols are not enforced.

**Fig. 1:**
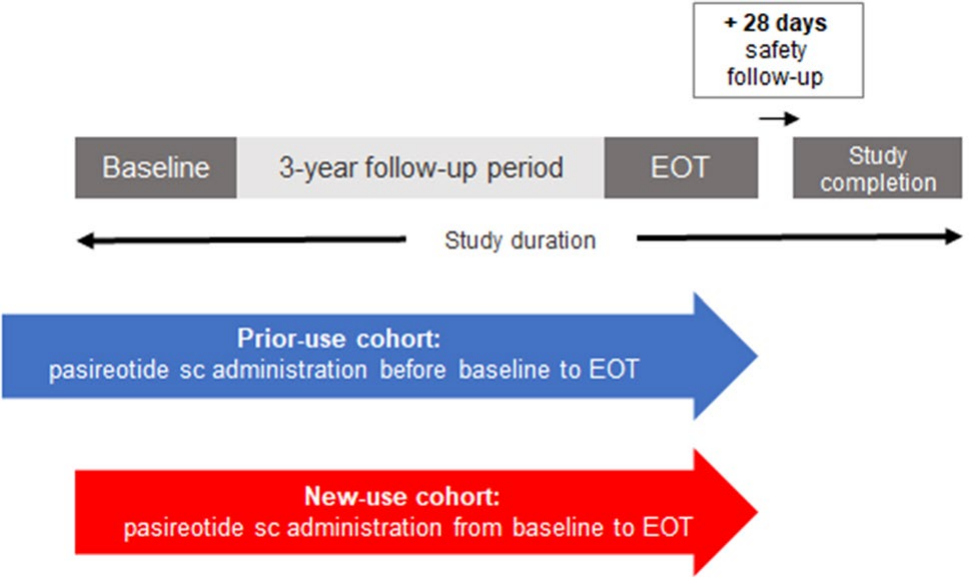
Study design. Patients discontinuing prematurely were followed up for 3 months after the last dose. *EOT* end of treatment, *sc* subcutaneous

Patients are treated with pasireotide according to the investigator’s judgment and in accordance with the local (country-specific) prescribing information. Enrolled patients are monitored for a period of up to 3 years from study entry. Patients who remain in the study for the planned 3-year observation period are monitored for an additional 28 days after this time for safety. In cases of premature study discontinuation, patients are monitored for an additional 3 months from the time of their last pasireotide dose, if feasible (Fig. 1).

### Objectives and assessments

The primary objective of the study is to document the long-term safety and tolerability of pasireotide (alone or in conjunction with other therapies) in patients with CD. The primary endpoint is the incidence of adverse events (AEs) and serious AEs (SAEs) related to pasireotide during the 3-year observation period. The secondary objective of the study is to document the short- and long-term efficacy of pasireotide, defined by the proportion of patients with a mean UFC (mUFC) level not exceeding the upper limit of normal (ULN) at 1, 3, 6, 12, 24 and 36 months after enrollment. mUFC is calculated according to local site procedures and usually as the average of two or three 24-h urine measurements.

Safety and tolerability were assessed by recording AEs at each visit. AEs are defined using the Medical Dictionary for Regulatory Activities v18.1. The severity of AEs was defined as mild (grade 1), moderate (grade 2), severe (grade 3) or life threatening (grade 4) according to the National Cancer Institute’s Common Terminology Criteria for AEs v3.0; hyperglycemia was graded according to fasting plasma glucose levels: > ULN to 160 mg/dL (grade 1), > 160–250 mg/dL (grade 2), > 250–500 mg/dL (grade 3), and > 500 mg/dL (grade 4). In addition to the standard criteria for SAE reporting [13], any observed AE that was listed as an important medical event by the European Medicines Agency was referred to the treating investigator and recorded as an SAE at their discretion, according to the requirements for non-interventional studies.

### Statistical methods

Safety analyses were performed on data from patients who received ≥ 1 dose of pasireotide and had ≥ 1 post-baseline safety assessment (safety analysis set). Efficacy analyses were performed on data from patients who received ≥ 1 dose of pasireotide (full analysis set); assessment of the proportion of patients with mUFC ≤ ULN at baseline and over time was based on the patients with an evaluable assessment at the given time point. Mean [95% confidence interval (CI)] change from baseline in mUFC and serum cortisol was calculated for patients with evaluable measurements at both time points.

Data are presented for the overall population and according to whether patients were receiving pasireotide before study entry (prior-use group) or initiated pasireotide at first study visit (new-use group). Results are reported as number (%) or mean [standard deviation (SD)] as appropriate. All analyses are descriptive in nature, and no formal comparisons between the prior-use and new-use groups have been performed. In the absence of a strict visit schedule, data are reported by visit windows.

The first patient visit was conducted on March 28, 2013 and, in accordance with the study protocol, the database is locked on a yearly basis to allow periodic assessment of efficacy and safety. The current analysis includes all data collected up to the most recent cut-off date of October 1, 2017.

## Results

### Study population

Between March 28, 2013 and October 1, 2017, 127 patients were enrolled in the study across 63 sites in 11 countries (Canada, Colombia, France, Germany, Israel, Italy, Lebanon, Netherlands, Romania, UK and USA). Individual patient numbers per study site ranged from 1 to 13. The majority of patients (96/127, 75.6%) were receiving pasireotide treatment before study entry, and the remainder (31/127, 24.4%) started pasireotide treatment at their first study visit (Table 1). Overall, 123/127 (96.5%) patients had at least one post-baseline safety assessment. Most (73.2%) patients had undergone prior pituitary surgery, while 22.8% of patients had received pituitary irradiation. Patient demographics were generally similar between the two groups of patients (Table 1). The proportion of females and patients with previous pituitary surgery and/or irradiation were numerically higher in the prior-use than in the new-use group.

**Table 1:** Patient characteristics at study baseline

	All patients N = 127	Prior-use n = 96	New-use n = 31
Mean age, years (SD)	49.9 (14.0)	50.0 (14.1)	49.6 (13.9)
≥65 years old, n (%)	20 (15.7)	15 (15.6)	5 (16.1)
Female, n (%)	101 (79.5)	79 (82.3)	22 (71.0)
Race, n (%)			
Caucasian	102 (80.3)	77 (80.2)	25 (80.6)
Black	1 (0.8)	1 (1.0)	0
Other	24 (18.9)	18 (18.8)	6 (19.4)
Mean time since diagnosis, months (SD)	73.5 (78.1)	83.4 (78.4)	42.8 (69.9)
CD status, n (%)			
De novo	23 (18.1)	11 (11.5)	12 (38.7)
Persistent/recurrent	100 (78.7)	81 (84.4)	19 (61.3)
Missing	4 (3.1)	4 (4.2)	0
Mean mUFC × ULN (SD)^a^	1.6 (1.7)	1.1 (1.0)	2.8 (2.5)
Previous pituitary surgery, n (%)^b^	93 (73.2)	74 (77.1)	19 (61.3)
Mean time since last pituitary surgery, months (SD)	61.2 (56.2)	65.3 (54.9)	45.4 (60.2)
Previous pituitary irradiation, n (%)	29 (22.8)	26 (27.1)	3 (9.7)
Mean time since last pituitary irradiation, months (SD)	57.1 (59.1)	57.0 (62.0)	57.7 (29.0)

^a^Mean mUFC values were based on 59 patients at baseline: 43 patients in the prior-use group and 16 patients in the new use group

^b^Seven patients in the prior-use group had details of last pituitary surgery missing

**Fig. 2:**
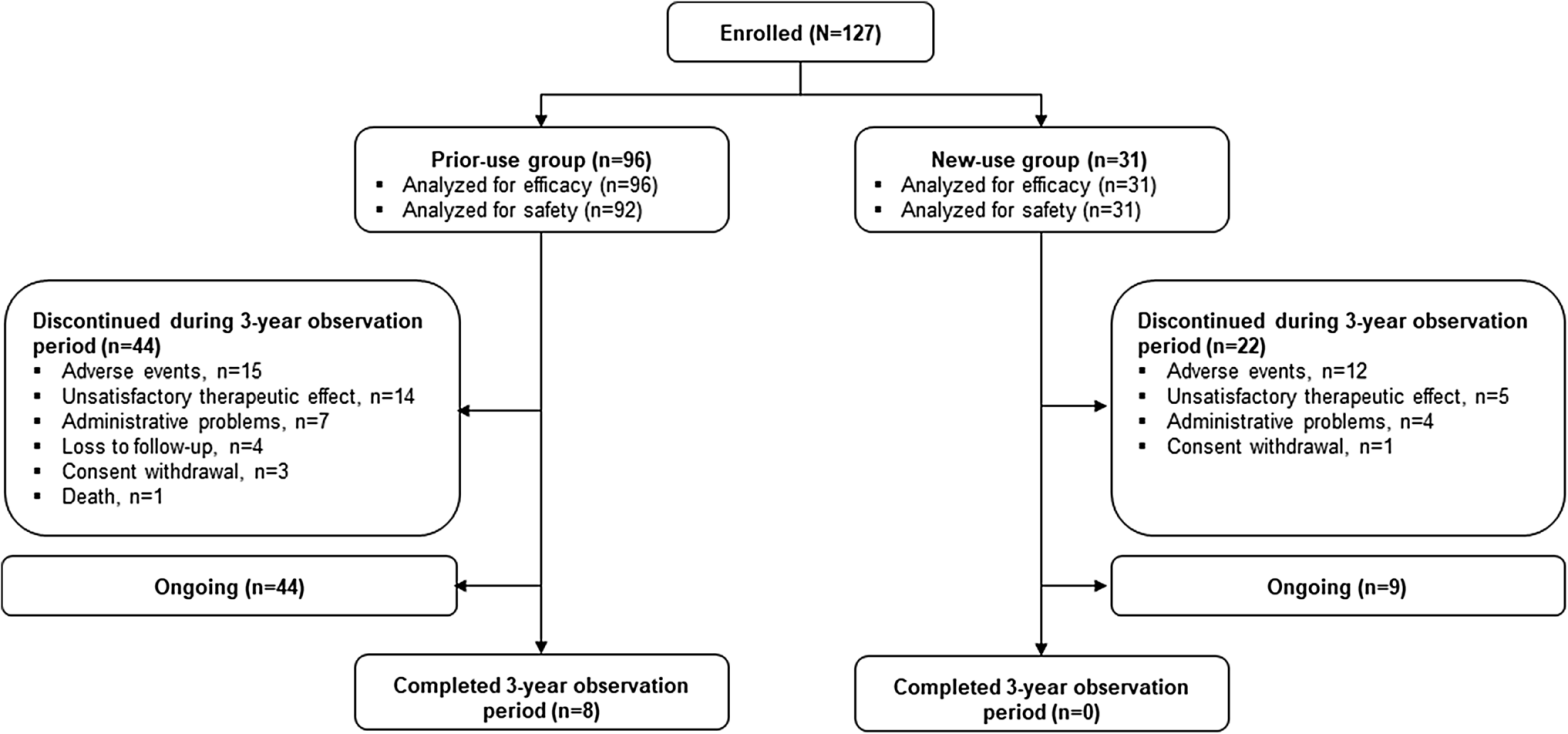
Patient disposition

At the time of data cut-off, 8/127 (6.3%) patients had completed the 3-year observation period, all of whom were in the prior-use group (Fig. 2). 53 (41.7%) patients were continuing to receive pasireotide in the study (new users: 9/31 [29.0%], prior users: 44/96 [45.8%]).

The median (range) duration of exposure to pasireotide was 19.1 months (0.1–118.4 [9.9 years]): 3.4 months (0.1–18.4) for new users and 30.5 months (1.9–118.4) for prior users, including prior treatment (on-study duration: 12.0 months [0.3–41.6]). Overall, 49/123 (39.8%), 20/123 (16.2%) and 4/123 (3.3%) patients, respectively, have received at least 12, 24 and 36 months of pasireotide treatment within the study. Including exposure prior to study start, 32/123 (26.0%) patients have received at least 36 months of pasireotide treatment. The median (range) average daily dose from study start was 1.2 mg/day (0.6–1.8) in the new-use group and 1.2 mg/day (0.2–1.8) in the prior-use group.

66 (52.0%) patients discontinued the study (new users: 22/31 [71.0%], prior users: 44/127 [45.8%]). The most common reasons for discontinuation in the new-use and prior-use groups were AEs (38.7% and 15.6%), unsatisfactory therapeutic effect (16.1% and 14.6%) and administrative problems (12.9% and 7.3%).

Most patients who remained on treatment at months 6 (77.0%, n = 57/74), 12 (76.0%, n = 38/50) and 24 (80.0%, n = 16/20) received pasireotide monotherapy for the duration of these treatment periods. Other drugs used in combination to treat CD, as reported by the investigators, included cabergoline (month 6: n = 5/74 [6.8%], month 12: n = 4/50 [8.0%], month 24: n = 1/20 [5.0%]) and metyrapone (month 6: n = 4/74 [5.4%], month 12: n = 2/50 [10.0%], month 24: n = 1/20 [5.0%]).

### Safety

From the start of the study, 98/123 (79.7%) patients experienced an AE. Overall, 61/123 (49.6%) patients experienced one or more drug-related AEs (i.e. AEs that were suspected by the investigator to be related to pasireotide treatment): 24/31 (77.4%) and 37/92 (40.2%) patients in the new-use and prior-use groups, respectively. Grade 3/4 drug-related AEs occurred in 18/123 (14.6%) patients: 7/31 (22.6%) patients in the new-use group and 11/92 (12.0%) patients in the prior-use group (Table 2).

**Table 2:** Most common drug-related AEs (≥ 5% in new-use or prior-use group)

Preferred term	All patients N = 123 n (%)		Prior-use N = 92 n (%)		New-use N = 31 n (%)	
	All grades	Grade 3 or 4	All grades	Grade 3 or 4	All grades	Grade 3 or 4
Total	61 (49.6)	18 (14.6)	37 (40.2)	11 (12.0)	24 (77.4)	7 (22.6)
Hyperglycemia-related AE						
Hyperglycemia	14 (11.4)	3 (2.4)	5 (5.4)	0	9 (29.0)	3 (9.7)
Diabetes mellitus^a^	9 (7.3)	2 (1.6)	6 (6.5)	1 (1.1)	3 (9.7)	1 (3.2)
Type 2 diabetes mellitus^a^	2 (1.6)	0	0	0	2 (6.5)	0
Nausea	17 (13.8)	2 (1.6)	7 (7.6)	0	10 (32.3)	2 (6.5)
Diarrhea	13 (10.6)	1 (0.8)	8 (8.7)	1 (1.1)	5 (16.1)	0
Cholelithiasis	7 (5.7)	0	4 (4.3)	0	3 (9.7)	0
Vomiting	6 (4.9)	0	3 (3.3)	0	3 (9.7)	0
Fatigue	6 (4.9)	0	3 (3.3)	0	3 (9.7)	0
Drug ineffective	5 (4.1)	1 (0.8)	5 (5.4)	1 (1.1)	0	0
Feeling cold	2 (1.6)	0	0	0	2 (6.5)	0
Hypertension	2 (1.6)	0	0	0	2 (6.5)	0

AEs are presented by preferred term. AEs related to hyperglycemia are grouped. Patients were counted in more than one row if they had separate AEs reported according to different terms (e.g. diabetes mellitus and type 2 diabetes mellitus)

^a^As there were no guidelines in this real-world study to define the term ‘diabetes mellitus’, its usage depended on the local criteria

The most common drug-related AEs (≥ 10% of patients overall) were nausea (17/123, 13.8%), hyperglycemia (14/123, 11.4%) and diarrhea (13/123, 10.6%), and these AEs were more commonly reported in new users than in prior users (Table 2).

Hyperglycemia-related AEs suspected to be related to pasireotide were reported in 29/123 (23.6%) patients: 14/31 (45.2%) patients in the new-use group and 15/92 (16.3%) patients in the prior-use group. A grade 3 hyperglycemia-related AE occurred in 5/123 (4.1%) patients (new users: 4/31 [12.9%], prior users: 1/92 [1.1%]); each of these patients had a recorded history of diabetes prior to entering the study. Gallbladder/biliary-related AEs were reported in 11/123 (8.9%) patients: 3/31 (9.7%) patients in the new-use group and 8/92 (8.7%) patients in the prior-use group. 2 (1.6%) patients, both of whom were prior users of pasireotide (2.2%), experienced a total of three grade 3 gallbladder/biliary-related AEs: increased blood alkaline phosphate, increased blood bilirubin, and cholecystitis. No grade 4 hyperglycemia- or gallbladder/biliary-related AEs (regardless of suspected drug relationship) were observed during the current reporting period.

Study-drug-related SAEs occurred in 17/123 (13.8%) patients (new users: 7/31 [22.6%], prior users: 10/92 [10.9%]). The most common drug-related SAEs were hyperglycemia (4/123, 3.3%), type 2 diabetes mellitus, cholelithiasis, and ineffective drug (all 2/123, 1.6%). Drug-related SAEs of hyperglycemia, type 2 diabetes mellitus and cholelithiasis were more common in the new-use group than in the prior-use group (Table 3). In total, seven patients experienced a hyperglycemia-related SAE considered to be related to study drug (grade 1/2, n = 4; grade 3, n = 3). Two of these hyperglycemia-related SAEs were reported concomitantly with SAEs of worsening of CD or lack of efficacy. There were no reports of diabetic ketoacidosis or hyperosmolar non-ketotic coma.

**Table 3:** SAEs suspected to be drug related (> 1% in new-use or prior-use group)

Preferred term	All patients N = 123 n (%)	Prior-use N = 92 n (%)	New-use N = 31 n (%)
Total	17 (13.8)	10 (10.9)	7 (22.6)
Hyperglycemia-related AE			
Hyperglycemia	4 (3.3)	1 (1.1)	3 (9.7)
Type 2 diabetes mellitus^a^	2 (1.6)	0	2 (6.5)
Diabetes mellitus^a^	1 (0.8)	1 (1.1)	0
Cholelithiasis	2 (1.6)	1 (1.1)	1 (3.2)
Drug ineffective	2 (1.6)	2 (2.2)	0
Nausea	1 (0.8)	0	1 (3.2)
Acute adrenocortical insufficiency	1 (0.8)	1 (1.1)	0
ACTH deficiency	1 (0.8)	1 (1.1)	0
Cholangitis	1 (0.8)	1 (1.1)	0
Cholecystitis	1 (0.8)	1 (1.1)	0
Post-procedural bile leak	1 (0.8)	1 (1.1)	0
Post-procedural complication	1 (0.8)	1 (1.1)	0
Benign pituitary tumor	1 (0.8)	1 (1.1)	0
Hypoesthesia	1 (0.8)	1 (1.1)	1 (0.8)
Presyncope	1 (0.8)	1 (1.1)	1 (0.8)

AEs are presented by preferred term. AEs related to hyperglycemia are grouped. Patients were counted in more than one row if they had separate AEs reported according to different terms (e.g. diabetes mellitus and type 2 diabetes mellitus)

^a^As there were no guidelines in this real-world study to define the term ‘diabetes mellitus’, its usage depended on the local criteria

Overall, 24/123 (19.5%) patients discontinued the study because of a drug-related AE: 10/31 (32.3%) and 14/92 (15.2%) patients in the new-use and prior-use groups, respectively. Of these, 3 (9.7%) patients in the new-use group and 4 (4.3%) in the prior-use group discontinued because of a drug-related SAE. The most common drug-related AEs leading to discontinuation were hyperglycemia (5/123 [4.1%]), ineffective study drug (5/123 [4.1%]) and nausea (4/123 [3.3%]). Greater proportions of patients in the new-use group than in the prior-use group discontinued because of hyperglycemia or nausea (Table 4).

**Table 4:** AEs suspected to be drug related leading to discontinuation (≥ 2% in new-use or prior-use group)

Preferred term	All patients N = 123 n (%)	Prior-use N = 92 n (%)	New-use N = 31 n (%)
Total	24 (19.5)	14 (15.2)	10 (32.3)
Hyperglycemia-related AE			
Hyperglycemia	5 (4.1)	2 (2.2)	3 (9.7)
Diabetes mellitus	2 (1.6)	1 (1.1)	1 (3.2)
Diabetic metabolic decompensation^a^	2 (1.6)	2 (2.2)	0
Blood glucose increased	1 (0.8)	0	1 (3.2)
Drug ineffective	5 (4.1)	5 (5.4)	0
Nausea	4 (3.3)	0	4 (12.9)
Glucocorticoid deficiency	2 (1.6)	2 (2.2)	0
Palpitations	1 (0.8)	0	1 (3.2)
Diarrhea	1 (0.8)	0	1 (3.2)
Feces pale	1 (0.8)	0	1 (3.2)
Flatulence	1 (0.8)	0	1 (3.2)
Gastrointestinal disorder	1 (0.8)	0	1 (3.2)
Vomiting	1 (0.8)	0	1 (3.2)
Fatigue	1 (0.8)	0	1 (3.2)
Feeling cold	1 (0.8)	0	1 (3.2)
Parosmia^b^	1 (0.8)	0	1 (3.2)
Irritability	1 (0.8)	0	1 (3.2)
Polyuria	1 (0.8)	0	1 (3.2)
Hypertension	1 (0.8)	0	1 (3.2)

AEs are presented by preferred term. AEs related to hyperglycemia are grouped. Patients were counted in more than one row if they had separate AEs reported according to different terms (e.g. diabetes mellitus and type 2 diabetes mellitus)

^a^Both patients with diabetic metabolic decompensation had an active medical history of diabetes mellitus at baseline and were reported to have a worsening of diabetes during pasireotide treatment. The AE resolved after initiation of insulin in one patient and discontinuation of pasireotide in the other patient

^b^Dysfunction concerning sense of smell

Four deaths, all in the prior-use group, were recorded after study start, none of which were considered by the investigator to be related to pasireotide. Two deaths occurred on treatment (i.e. up to 28 days after discontinuation of study treatment), one of which was attributed to pneumonia in a 66-year-old female who had a history of hypertension, sleep apnea, diabetes mellitus and obstructive pulmonary disease, and the other to metastatic pituitary cancer with worsening of hypercortisolism, pituitary tumor growth and kidney insufficiency in a 37-old-year male. Two patients died during post-treatment follow-up: one from a cerebrovascular accident, occurring 67 days after the last dose, in a 65-year-old male with a history of hypertension, hyperlipidemia and diabetes mellitus; and the other attributed to pneumonia 51 days after the last dose in a 77-year-old male with a history of diabetes mellitus and coronary heart disease.

### Efficacy

**Fig. 3:**
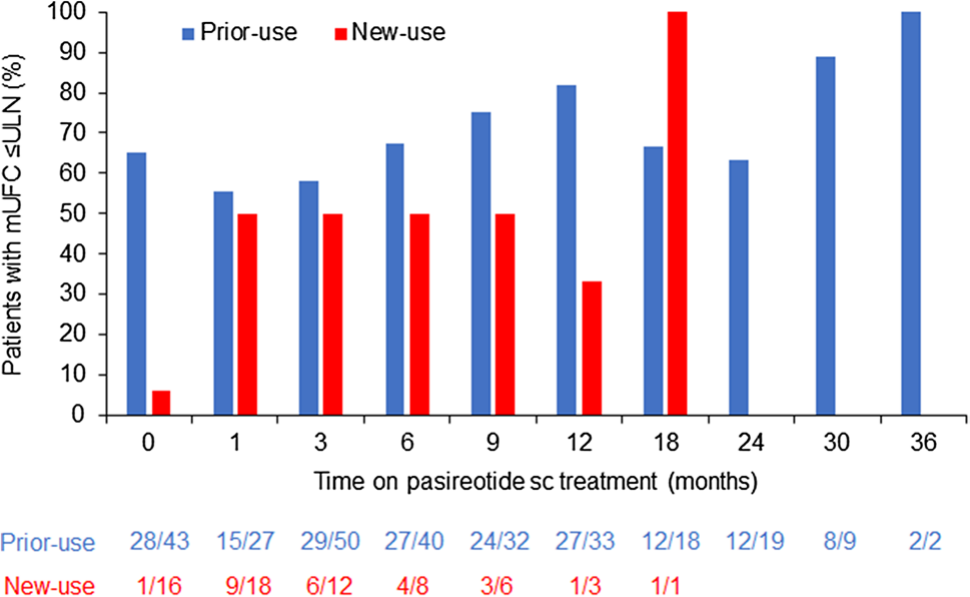
Proportion of patients with mUFC ≤ ULN. Numbers beneath the graph show number of evaluable patients with mUFC ≤ ULN

mUFC assessments were available for 59/127 (46.4%) patients at baseline: 16/31 (51.6%) and 43/96 (45.0%) patients in the new-use and prior-use groups, respectively. In the prior-use group, 28/43 (65.1%) patients with an evaluable assessment had mUFC ≤ ULN at baseline. The high proportion of patients with evaluable assessments who had mUFC ≤ ULN was maintained over time: 27/40 (67.5%), 27/33 (81.8%) and 12/19 (63.2%) patients at months 6, 12 and 24, respectively. Two patients had evaluable mUFC assessments at the end of the 3-year observation period (month 36), both of whom had mUFC ≤ ULN (Fig. 3).

At baseline, mean (SD) mUFC level for patients in the prior-use group was 1.1 × ULN (1.0, n = 43) and remained stable over the course of the study in patients with evaluable assessments: 1.2 × ULN (1.4, n = 40; mean change [95% CI] + 0.2 × ULN [− 0.3, 0.3]) at month 6, 0.7 × ULN (0.5, n = 33; mean change 0.0 × ULN [− 0.2, 0.2]) at month 12, and 0.8 × ULN (0.5, n = 19; mean change − 0.1 × ULN [− 0.4, 0.2]) at month 24. Mean (95% CI) change in serum cortisol was 13.7 nmol/L (− 3.8, 99.3; n = 19) at month 6, − 7.0 (− 129.7, 69.5, n = 15) at month 12, and − 51.1 nmol/L (− 35.0, 41.0, n = 6) at month 24.

In the new-use group, 1/16 (6.3%) patients with an evaluable assessment had mUFC ≤ ULN at baseline. For patients with evaluable assessments, normal mUFC levels were achieved by 9/18 (50.0%), 6/12 (50.0%), 4/8 (50.0%) and 3/6 (50.0%) patients at months 1, 3, 6 and 9, respectively, after which few patients had an evaluable mUFC assessment (Fig. 3).

For patients in the new-use group, mean (SD) mUFC level at baseline was 2.8 × ULN (2.5, n = 16), which decreased to 1.5 × ULN (1.3, n = 18; mean change [95% CI] − 1.8 × ULN [− 3.8, 0.5]) at month 1, 2.2 × ULN (3.8, n = 12; mean change − 1.6 × ULN [− 3.6, 0.1]) at month 3, and 1.1 × ULN (0.8, n = 8; mean change − 1.4 × ULN [− 4.0, 0.6]) at month 6. Mean (95% CI) change in serum cortisol was − 171.1 nmol/L (− 656.6, 80.3; n = 7) at month 1, − 26.3 (− 477.3, 193.1; n = 5) at month 3, and 8.1 nmol/L (493.9, 311.8; n = 5) at month 6.

## Discussion

The interim results from this international real-world study support the favorable long-term benefit–risk profile of pasireotide in patients with CD, which has been previously demonstrated in a Phase III clinical trial [10, 14]. The primary objective of this ongoing study is to document the long-term safety and tolerability profile of pasireotide in patients with CD in a real-world setting. Notably, more than 25% of patients who participated in our study had received over 3 years of treatment with pasireotide when including treatment exposure prior to study entry (maximum treatment duration of 9.9 years).

Half (50%) of the patients experienced a drug-related AE and 14% of patients experienced a drug-related SAE, the primary endpoint of the study, from study start. Most (70%) drug-related AEs were mild to moderate in severity and were consistent with the known safety profile of pasireotide [9], the most common being hyperglycemia-related AEs, nausea and diarrhea. AEs and SAEs were less common in patients who were treated with pasireotide prior to study entry than in those who initiated pasireotide at first study visit, and fewer prior users of pasireotide discontinued because of AEs than new users (15% and 32%, respectively). The lower rate of drug-related AEs and SAEs in prior versus new users may be explained by a potential selection bias: patients who tolerated pasireotide were more likely to continue pasireotide treatment (prior-use group). Furthermore, the most common AEs generally occur shortly after initiation of therapy.

Gastrointestinal AEs are a known class effect of somatostatin analogues. During our real-world study, a higher proportion of patients in the new-use group experienced AEs of nausea (32% vs. 8%) and diarrhea (16% vs. 9%) compared with the prior-use group, potentially reflecting an improvement in gastrointestinal tolerance over time. Compared with the previous Phase III study of twice-daily pasireotide in previously pasireotide-naïve patients, fewer patients in the new-use group of our study had a recorded AE of nausea (32% vs. 52%) or diarrhea (16% vs. 58%) [9]. The proportion of patients who discontinued because of any AE was slightly higher in the new-use group in our real-world study compared with the 12-month Phase III study (32% vs. 16%). Differences in the frequency of AEs and safety-related discontinuation rates between the two studies may result from numerous factors, including differences in duration of treatment/observation, the higher frequency of scheduled visits and safety assessments in the clinical trial setting, and differences in patient populations.

Hyperglycemia-related AEs have been frequently observed in clinical studies of pasireotide (57–77% of patients) [9, 15–17]. Increases in blood glucose levels predominantly occur soon after initiation of pasireotide, are more common in patients with pre-existing diabetes mellitus or impaired glucose tolerance, and can be effectively managed with appropriate antidiabetic medication soon after onset [9, 18]. Hyperglycemia associated with pasireotide results from the suppression of insulin secretion and decreased incretin response and is reversible upon treatment discontinuation [18–20]. In our study, hyperglycemia-related AEs that were suspected to be related to study drug were reported in < 25% of participating patients, and in a greater proportion of new users than prior users of pasireotide (45% and 16%, respectively). In addition, few patients experienced grade 3 hyperglycemia-related AEs, which were almost exclusively limited to new users (13% vs. 1%; no patients experienced a grade 4 event). Each patient who experienced a grade 3 hyperglycemia-related AE during the study had a prior history of diabetes. Results from this study indicate that pasireotide-associated hyperglycemia can be effectively managed with appropriate antidiabetic treatment in the clinical practice setting, as shown by the low rate (4%) of patients in this real-world evidence study discontinuing for this reason. The lower occurrence of hyperglycemic events in prior-use patients may have resulted from the appropriate management of pasireotide-associated hyperglycemia with antidiabetic agents prior to enrollment, as well as the improvement in hypercortisolism. In addition, patients with uncontrolled hyperglycemia on pasireotide may have been less likely to continue treatment as part of the study. It is important that blood glucose levels are monitored soon after the start of pasireotide treatment so that appropriate intervention can be taken promptly if these levels rise [21]. As decisions on the management of hyperglycemia were made by the local investigator, it was not possible to assess the effect of specific therapeutic approaches on blood glucose levels in this real-world study. A Phase IV study is currently ongoing to evaluate the effects of incretin-based therapy compared with insulin on glycemic control at 16 weeks in patients with CD or acromegaly (http://ClinicalTrials.gov NCT02060383).

In a previous Phase III study, pasireotide provided rapid and sustained reductions in mUFC and normalized levels in ~ 20% of enrolled patients with CD at months 6 and 12 [9]. In this ongoing real-world study, normal mUFC levels were achieved by 50% of new users who had an evaluable assessment within the first month of pasireotide treatment. Importantly, this high response rate was maintained for up to 9 months of treatment; however, few of these patients had evaluable assessments after this point. Future analyses from this study should allow us to explore the long-term response to pasireotide in new-use patients. Direct comparison of response rates for new-use patients in our study and the previous Phase III trial is not possible. Long-term control of mUFC levels was demonstrated by a significant number of patients who were receiving pasireotide prior to study start: 65% of these patients had mUFC ≤ ULN at baseline, compared with 82% and 67% of patients after a further 12 and 24 months of treatment, respectively. It is important to note that response rates are reported for patients with evaluable assessments in this study. As a result, response rates may be overstated as patients who discontinued treatment because of an unsatisfactory therapeutic effect (16% and 15% of patients in the new- and prior-use groups, respectively) were not included in the analyses. Although patients were permitted to receive concomitant medications for CD (most commonly cabergoline), the majority of patients (76% at month 12 and 80% at month 24) received pasireotide as monotherapy during the study. The higher response rates seen for the prior-use than the new-use group likely reflect the fact that patients with a favorable response to pasireotide would be more likely to continue receiving treatment in this study.

While resection of the ACTH-secreting pituitary adenoma by transsphenoidal surgery is first-line treatment for most patients with CD [5], multimodal treatment is frequently required in order to control hypercortisolism in patients with persistent or recurrent disease [1, 5]. In this real-world study, almost one-quarter of patients had received prior pituitary irradiation, which is generally considered third-line treatment in CD [22]. Interestingly, fewer patients had received prior pituitary irradiation in the new-use group (10%) than in the prior-use group (27%). As patients in the new-use group generally had a more recent diagnosis of CD than patients in the prior-use group (mean time since diagnosis: 43 vs. 83 months, respectively), it is possible that patients and physicians have been more willing in recent years to use medical therapy over radiotherapy for patients in whom surgery has failed, is not feasible or has been refused.

Our study has several limitations that we acknowledge. Owing to its observational nature, a therapy protocol and visit schedule are not imposed and, as such, specific efficacy assessments and study visits are not mandatory. So far, this has resulted in almost half of the patients who entered our study not having mUFC assessments at baseline and many having missing values at subsequent time points. In addition, some patients received concomitant medications for CD during this real-world study. Taken together, these factors may have complicated overall data interpretation. For the same reason, an accurate report of changes in clinical signs of hypercortisolism and health-related quality of life was not formally performed in the study. Nevertheless, a key strength of this study is the large number of patients receiving pasireotide therapy and monitored in real-world clinical practice.

In conclusion, the interim results from this observational study demonstrate that pasireotide provides rapid and sustained control of mUFC and offers acceptable tolerability in real-world clinical practice, with no new safety signals emerging over time. Notably, a lower incidence of hyperglycemia was observed in prior- compared with new-use patients, suggesting that pasireotide-associated hyperglycemia did not worsen over time if appropriately managed after onset. These findings support pasireotide as a long-term therapeutic option that can be utilized to provide sustained control of hypercortisolism for patients with CD.

## Data Availability

Novartis is committed to sharing with qualified external researchers access to patient-level data and supporting clinical documents from eligible studies. These requests are reviewed and approved by an independent review panel on the basis of scientific merit. All data provided are anonymized to respect the privacy of patients who have participated in the trial, in line with applicable laws and regulations. This trial data availability is done according to the criteria and processes described at www.clinicalstudydatarequest.com.
